# Reconstructing evolutionary trees in parallel for massive sequences

**DOI:** 10.1186/s12918-017-0476-3

**Published:** 2017-12-14

**Authors:** Quan Zou, Shixiang Wan, Xiangxiang Zeng, Zhanshan Sam Ma

**Affiliations:** 1 School of Computer Science and Technology, Tianjin University, Tianjin, People’s Republic of China; 2 Guangdong Province Key Laboratory of Popular High Performance Computers, Shenzhen University, Shenzhen, China; 3 State Key Laboratory of Genetic Resources and Evolution, Kunming Institute of Zoology, Chinese Academy of Sciences, Kunming, China; 4 Department of Computer Science, Xiamen University, Xiamen, China

**Keywords:** Evolutionary tree, Computational biology, Multiple sequence alignment, Algorithm, Hadoop, Spark

## Abstract

**Background:**

Building the evolutionary trees for massive unaligned DNA sequences is challenging and crucial. However, reconstructing evolutionary tree for ultra-large sequences is hard. Massive multiple sequence alignment is also challenging and time/space consuming. Hadoop and Spark are developed recently, which bring spring light for the classical computational biology problems. In this paper, we tried to solve the multiple sequence alignment and evolutionary reconstruction in parallel.

**Results:**

HPTree, which is developed in this paper, can deal with big DNA sequence files quickly. It works well on the >1*GB* files, and gets better performance than other evolutionary reconstruction tools. Users could use HPTree for reonstructing evolutioanry trees on the computer clusters or cloud platform (eg. Amazon Cloud). HPTree could help on population evolution research and metagenomics analysis.

**Conclusions:**

In this paper, we employ the Hadoop and Spark platform and design an evolutionary tree reconstruction software tool for unaligned massive DNA sequences. Clustering and multiple sequence alignment are done in parallel. Neighbour-joining model was employed for the evolutionary tree building. We opened our software together with source codes via http://lab.malab.cn/soft/HPtree/.

## Background

The reconstruction of evolution trees and alignments for large data are still open challenges for bioinformatics researchers [1, 2]. The third generation sequencing techniques promoted massive metagenome sequences, which call for OTU clustering and taxonomic labelling [3]. Besides deep understanding on the genes, population, species evolutionary relationships, evolutionary tree reconstruction also benefits for the metagenome and microbial genomics research.

Evolutionary tree reconstruction could be divided into three different situations. The first one is different species genomes evolutionary relationship reconstruction, which considers the influence from horizontal gene transfer [4, 5], incomplete lineage sorting [6, 7], gene orders with insertions and deletions [8], and rearrangement [9]. The second situation considers different homologous genes [10], where the maximum likelihood method is usually chosen for their perfect mathematical explanation [11]. Some researchers considered that networks could represent the evolutionary process better than trees [12]. The third one is to analyze the evolutionary relationships among the individuals in a population. In this case, massive similar sequences should be handled, and computer memory limitation often becomes the bottleneck.

Multiple sequence alignment is necessary for evolutionary tree software tools, including MEGA [13], MAFFT [14], SATe-II [15], IQ-TREE [16], iGTP [17], FastTree [18], and phangorn [19]. Most multiple sequence alignment tools cannot deal with massive sequences (eg. >10,000 sequences). Therefore, evolutionary tree reconstruction independent of multiple sequence alignment was developed [20], which is called the next-generation phylogenomics [21]. The divide-and-conquer algorithm [22] and distance model [23] have been employed, and the sequence distance was be computed according to different types of kmers [24, 25], word frequencies [26] or average common substrings [27]. They can avoid the time cost from pairwise sequence alignment. However, the performance would be decreased [28]. Due to the lack of evolutionary reconstruction tools together with multiple sequence alignment for massive unaligned sequences, it is essential and necessary to solve this problem with latest parallel computation techniques.

Some parallel techniques were tested for evolutionary tree, including multi-cores [29, 30], MPI [31–33], grid computing [34], GPU [35, 36], etc. However, there are no related references on the Hadoop and Spark platform. Hadoop has been utilized in multiple sequence alignment for handling large scale data in our previous work [37]. Here we build the evolutionary tree for massive unaligned DNA sequences with Hadoop and Spark framework.

## Results

### Data

TreeBase [38] was selected as the golden benchmark in most of the current evolutioanry reconstruction software tools. But the data from TreeBase are rather small. We try to solve the massive sequences problem, so TreeBase is not suitable in this work. Since there is no large scale benchmark datasets, we only selected running time as the performance measurement.

Human mitochondrial genomes [39] and 16S rRNAs [40] were employed for testing in our work. There are 672 human mitochondrial genomes in the human mitochondrial genomes dataset. In order to test the “big data” performance, the data were duplicated 20, 50, and 100 times separately. In these datasets, sequences were similar. We also tested the performance in the 16S rRNAs datasets, in which sequences have low similarity. There are two differnt files. The first file is 156 *MB*, while the second is 1.4 *GB*. All the datasets seemed more bigger than TreeBase.

### Comparison with the state-of-the-art software tools

We have tried our datasets with MEGA [13], MAFFT [14], SATe-II [15], RAxML [33], STELLS [41], MrBayes [30], Beagle [42], Beast [43], and PLL [31]. However, most of them cannot even handle the smallest dataset. So we only compare the performance with RAxML, Phangorn, STELLS and IQ-Tree [16]. All these three software tools need the aligned files as the input. So we firstly emloyed HAlign [44] for multiple sequence alignment before the evolutionary trees reconstruction.

**Fig. 1:**
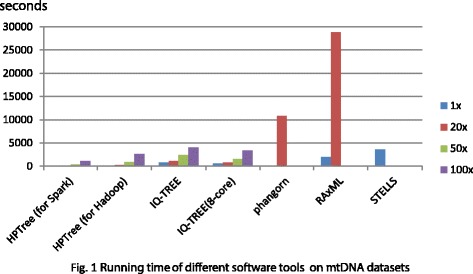
The running time on mt genome datasets with different numbers of Hadoop nodes. Running time of different software tools on mtDNA datasets

The running time was compared and showed in Fig. 1. The initial human mitochondrial genomes dataset is about 10 *MB*. After the duplicating, the 100× file is more than 1 *GB*. Since other tools can only work on single node, all the software tools were testing in one computer instead of the cluster. In Fig. 1, it seemed that HPTree outperform other tools even on a single node. Phangorn, RAxML and STELLS could handle the 1× file but cannot deal with the larger files.

**Fig. 2:**
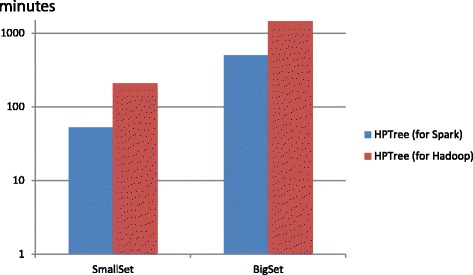
The running time on mt genome datasets with different numbers of Spark nodes. Running time with HPTree on 16S rRNA datasets

For the massive high-similarity DNA sequences, HPTree can build the evolutionary tree for over 1 *GB* file in several minutes. Then we tested the performance with low-similarity sequences. In our testing experiments, only HPTree could handle the two 16S rRNA datasets. The consuming time was shown in Fig. 2. For the low-similarity datasets, HPTree still works for the more than 1*GB* files.

### Speed up of the parallel mechanism

Single node performance of HPTree was shown in Figs. 1 and 2. Here we employed Hadoop on 2–4 nodes, and showed the performance of HPTree in clusters. Table 1 showed the running time of different nodes comparison. The parallel efficiency and the speed-up ratio of HPTree with Hadoop and Spark can be viewed from Table 1. The multi-codes cluster would accelerate and benefit for the massive big files. Besides, we can see that Spark platform has a better average performance than native Hadoop, which owes to the memory computing technology in Spark. For Hadoop MapReduce operator, all intermediate data will be saved in hard disk for disaster recovery, which is suitable for massive data processing but reduces the efficiency of programs. For Spark platform, intermediate data will be saved in memory as much as possible for reiterative computing, and the rest of intermediate data will be saved in hard disk [45]. Hence, our experiment result shows that Spark accelerate HPTree more remarkably than Hadoop.

**Table 1:** The running time of differen nodes comparison on human mitochondrial genomes dataset (Unit: seconds)

	1×	20×	50×	100×
4-nodes(Hadoop)	72	198	988	2657
3-nodes(Hadoop)	110	324	1631	3487
2-nodes(Hadoop)	157	494	2235	5384
4-nodes(Spark)	27	65	423	1095
3-nodes(Spark)	35	96	765	1770
2-nodes(Spark)	67	189	1232	2586

### Performance on the unaligned sequences

We have employed Halign for multiple sequence alignment as preprocess in the above testing. The most important point of HPTree is the ability of handling unaligned sequences, which is the key advantage beyond other evolutionary reconstruction tools. Multiple sequence alignment for massive sequences is also challenging and time/space consuming. HPTree also deals with this problem in parallel.

Tables 2 and 3 shows the running time of aligned and unaligned data on Hadoop and Spark platform, respectively. Our bigdata sets, including human mitochondrial genomes and 16S rRNAs, were both tested. Because no other software tool could deal with the unaligned sequences, we just show the expresiment results of HPTree. Tables 2 and 3 showed several interesting results. We conclude that HPTree runs observably faster on Spark platform than on Hadoop platform. As the sequence number grows larger, the multiple sequence alignment would occupy littler in the total running time. In the 1× and 20× datasets, running time increased sharply for the unaligned sequences. But in the 50×, 100× and 16 s rRNA datasets, which contain more than 10, 000 sequences, multiple sequence alignment would not influence the time performance sharply. Neighbour joining occupied most of the running time. It suggests that multiple sequence alignment is not the only problem for massive sequences, but the tree topology and branch distance computation is also the key challenge.

**Table 2:** The running time of human mitochondrial genomes datasets between aligned and unaligned sequences (Unit: seconds)

	1×	20×	50×	100×
Unaligned(Hadoop)	213	851	1722	4365
Aligned(Hadoop)	72	198	868	2657
Unaligned(Spark)	56	238	846	1720
Aligned(Spark)	27	65	423	1095

**Table 3:** The running time of 16S rRNA datasets between aligned and unaligned sequences (Unit: seconds)

	Small	Big
Unaligned(Hadoop)	15,736	106,400
Aligned(Hadoop)	12,464	86,400
Unaligned(Spark)	4739	35,869
Aligned(Spark)	3159	30,012

### Comparison with HPTree on Hadoop and spark

Hadoop and Spark are popular distributed computing frameworks. Fault-tolerant for the former relied on HDFS system based on backups on hard disk. Such a design is suitable for ultra-large dataset (larger than TB class) because memory is not able to load such ultra-large dataset. Facts proved that Hadoop framework has achieved great success on the distributed computing field based on MapReduce programming model. Fault-tolerant for Spark relied on RDD data structure based on backups on memory and hard disk. From the above experiment result, efficient reiterative computing on HPTree for Spark runs faster than HPTree on Hadoop. However, Spark is closely associated with Hadoop, and HDFS system would not be replaced. Ultra-large dataset need to be persisted on Hadoop HDFS system based on hard disk and need to be efficient computed on Spark MapReduce framework. Our experiments show that Spark and HDFS system can cope with ultra-large multiple sequence alignment and evolutionary analysis issue.

## Discussion

Evolutionary tree reconstruction for massive unaligned sequences is still an open challenge. In this paper, we employed the Hadoop and Spark platform for the massive DNA sequences evolutionary relationship analysis in parallel. In order to decrease the running time, the neighbour-joining model is chosen for the tree building. Different from the common neighbour-joining algorithm, sequence clustering is done first and multiple sequence alignment and sub-evolutionary tree construction are excuted in parallel on each node. The final tree is built combining the subtree results. HPTree could handle the unaligned massive sequences, while the state-of-arts tools cannot. In the more than 1 *GB* files experiments, HPTree works well on both high and low similarity sequences.

In this work, edit distance is chosed to measure the evolutionary distance because of simplicity and speed. Indeed, more complex evolutionary distance models should be considered, which is the future work. A smarter data structure on edit distance [46, 47] would facilitate the acceleration of parallel computing, which is also an opportunity for future work and improvement.

RNA is viewed similarly to DNA in this work. The RNA secondary structure information [48–51] is not considered in the alignment or involved in the evolutionary tree built by HPTree. RNA clustering or evolutionary analysis always requires secondary structure or base pair-matching information, such as microRNA family annotation in the miRBase [52] and RFam database [53, 54]. Therefore, a Hidden Markov Model is always employed in the alignment and distance computing process, but it is rather time consuming and unsuitable for ultra-large data.

Moreover, evolutionary networks are superior to trees for large-scale and complex evolutionary analysis. Our parallel strategy also suits network reconstruction. However, this approach is somewhat complicated and will be undertaken in the future.

## Methods

Although MapReduce frame was employed in HPTree similar to multiple sequence alignment, the core techniques were totally different from HAlign. The main core problem is the subtree partition, which involved the load balancing in each node in the next step. Here we also employed MapReduce to clustering the massive sequences firstly. If some clusters are more bigger or smaller than others, we will split the big clusters and combine the small ones. Related techniques were introduced in our previous work [55, 56].

After clustering, we chose neighbour-joining (NJ) model for the subtree construction. Comparing with maximum parsimony and maximum likelihood models, neighbour-joining is fast and least time/space consuming. Maximum parsimony and maximum likelihood models are both complex and not suitable for the MapReduce frame.

The major advantage of HPTree is the ability for unaligned sequences. We employed Hadoop and Spark for the multiple sequence alignment in the preprocess. Then we introduced the detailed process respectively.

### Multiple sequence alignment with Hadoop

We aimed at the ultra-large scale data. So in every step we selected the simplest model and method. Here center star multiple sequence alignment algorithm was chosen instead of tree based alignment algorithm. In center star algorithm, “centre sequence” is chosen as a standard one, and every other sequence would be aligned with the “centre sequence” pairwisely. After the pairwise alignments, all the spaces inserted to the “centre sequence” would be summed up, and the other sequences will be supplied the corresponding spaces. Finally, all the sequences will have the same length. This is the whole process of the center star multiple sequence alignment.

We employ Hadoop to accelerate this process in parallel. So all the sequences would be divided into several parts. In order to save time, we randomly selected a sequence as the “centre sequence”.

It is known that the entries in Map Reduce are recorded using the (key, value) format. We use key to denote the sequence name and value for the DNA sequence. Before the parallel computing, we pre-process the input sequences and delete the illegal characters and strange sequences. Then, all the input sequences are formatted as (key, value) pairs for Hadoop. As all the sequences are similar, the first sequence is selected as the centre sequence.

In the first stage of the Map function, the data file is divided automatically into several split files of size 64 MB or less. These split files are sent to different data nodes and aligned to the centre sequence in parallel. After the alignment, the centre sequence and the sequence in the split file are updated with inserted spaces. They are still recorded using the (key, value) format, where the key is the sequence name and the value is the updated two aligned sequences. Then, the output (key, value) pairs enter the Reduce stage.

In the first stage of the Reduce function, data are not processed and are output to the HDFS file system directly. The data are then collected from the HDFS file system to a local computer, and the aligned centre sequences are extracted and gathered. For the *n* aligned centre sequences, we count the maximum spaces between every two neighbouring characters. The maximum spaces are retained for the Final Centre Sequence.

The second Map-Reduce phase is similar to the first stage. All the aligned sequences in the first stage are aligned again to the Final Centre Sequence. As the Final Centre Sequence has the maximum spaces between all characters, there will be no spaces inserted into the Final Centre Sequence. Thus, all the other sequences will be aligned to the same length with the Final Centre Sequence, producing the final alignment result. Indeed, the original centre star method records the inserted space positions for the Final Centre Sequence instead of the second alignment. However, when handling massive data, the distributed storage of the records is a problem. As the DNA sequences are similar, the k-band alignment is linearly time consuming. Thus, the second Map-Reduce alignment is employed.

### Multiple sequence alignment with spark

Hadoop mainly contains Hadoop Distributed File System (HDFS) for distributed storage and MapReduce programming model for big datasets. HDFS stores data on inexpensive machines, providing dependable fault-tolerant mechanism and high-aggregate bandwidth across clusters. Spark aims to blueprint a programming model that extends applications of MapReduce model and achieves high computational efficiency-based memory cache.

**Fig. 3:**
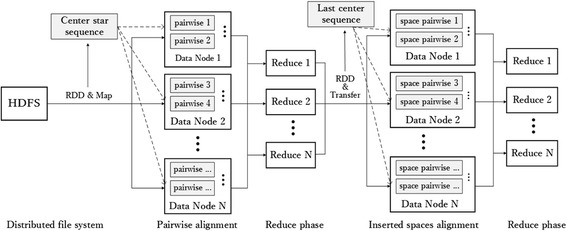
MSA procedures based on Spark distributed framework

Spark designs an abstract data structure named resilient distributed datasets (RDDs) to support efficient computing and to ensure distribution of datasets on cluster machines. RDDs staying in memory cache will visibly reduce load time when requiring replication, especially in iterative operations. From Fig. 3, to further reduce time and cost, two types of operations in RDDs are designed: transforms and actions. Transforms only deliver computing graphs, which only describe how to compute and not how to carry out computing operations, such as map and filter operation. Actions carry out computing, such as reduce and collect operations, results of which are stored as new RDDs. Based on these operations, RDDs are efficiently executed in parallel. To ensure dependable fault tolerance, RDDs will be recomputed after data loss, for example, because of halting of individual machines. Based on RDDs, Spark can implement up to 100 times theoretical speed than Hadoop in real-world datasets.

As mentioned before, based on parallel computing, we first cluster all MSA results into several clusters. Then, we calculate individual phylogenetic tree based on individual clusters. Last, all phylogenetic trees are merged on clusters into the final evolution tree. The approach comprises two key steps: initial clustering and MSA. MSA methods are determined by trie trees algorithm for similar nucleotide sequences. Then, we highlight the initial clustering procedure. Approximately 10% of all sequences are selected by random sampling for initial clustering. Then, functional distance of each pairwise sequence is calculated, clustered, and labeled until all sequences are identified. When few clusters whose number of elements is over 10%, then they are merged into other clusters; otherwise, they are divided into more balanced clusters until balanced construction. The entire procedure is designed for Spark parallel model.

### Implementation

HPTree is licensed under the GPL license and is implemented using Java, which can work on multiple operation systems. Hadoop 2.0 and Spark 2.0 are required for the parallel tool. We have constructed the web site http://lab.malab.cn/soft/HPtree/ for sharing the data, codes and software tools. A friendly web server is also developed. Users with just internet browser could draw the evolutionary trees by uploading their zipped fasta files.

## Conclusions

In this paper, we accelerate the evolutionary tree reconstruction with Hadoop and Spark. The ability of handling big data is also improved, especially unaligned sequences would be dealt with. Besides evolutionary analysis, the tree would also benefit for several other applications, such as DNA/protein sequence representation [57].

It can be anticipated that the proposed computational pipeline will have many potential applications. The multiple sequence alignment is one of the key techniques in biological sequence analysis. The proposed methods are able to efficiently reduce the computational cost, and therefore, they would be applied to protein, RNA, and DNA sequence analysis [58]. Recently, some algorithms have been proposed to extraction the evolutionary information from multiple sequence alignments, such as Pse-Analysis [59], and pseudo proteins [60]. Future studies will focus on extracting features from the evolutionary information.
